# Endoscopic Endonasal Transclival Approach to Ventral Pontine Cavernous Malformation: Case Report

**DOI:** 10.3389/fsurg.2021.654837

**Published:** 2021-05-12

**Authors:** Xiao Dong, Xiaoyu Wang, Anwen Shao, Jianmin Zhang, Yuan Hong

**Affiliations:** ^1^Department of Neurosurgery, The Second Affiliated Hospital, School of Medicine, Zhejiang University, Hangzhou, China; ^2^Brain Research Institute, Zhejiang University, Hangzhou, China; ^3^Collaborative Innovation Center for Brain Science, Zhejiang University, Hangzhou, China

**Keywords:** cavernous malformation, ventral pontine, endoscopic, endonasal, transclival

## Abstract

Ventral medial pontine cavernous malformations are challenging due to the location in eloquent tissue, surrounding critical anatomy, and potential symptomatic bleeding. Conventional approaches, such as anterolateral, lateral and dorsal approach, are associated with high risk of deleterious consequences due to excessive traction and damage to the surrounding tissues. The authors present an endoscopic endonasal approach for the resection of midline ventral pontine cavernous malformations, which follows principles of optimal access to brainstem cavernous malformations as the “two-point method.” No CSF leak or any other complications are obtained. The successful outcomes indicate that an individualized approach should be chosen before the surgery for brainstem cavernous malformations. With the advance of techniques, endoscopic endonasal approach could provide the most direct route to ventral pontine lesions with safety and efficiency.

## Highlights

Ventral medial pontine cavernous malformations are challenging. Conventional approaches are associated with high risk of complications. The authors present an endoscopic endonasal approach for the resection of midline ventral pontine cavernous malformations, with no CSF leak or any other complications obtained.

## Introduction

Brainstem cavernous malformations (BSCMs), characterized as low-flow, low pressure abnormally dilated vascular channels lined by a single layer of endothelium, accounts for 9~35% of all intracranial cavernous malformations (1). They occur mostly in the pons rather than in the midbrain and medulla, occasionally associated with venous malformations. When hemorrhages occur, BSCMs tend to demonstrate acute focal neurological deficits. While some of them have a tendency of spontaneous remission, others may cause severe and permanent neurological dysfunction when the eloquent area of brainstem is involved. It is reported in the published literature that the annual bleeding rate of BSCMs is 2.3~10.6%. And the prior hemorrhage is a significant predictor of rebleeding, with the rate increase to 5.0~21.5% (2).

Surgical intervention is recommended in patients who have experienced ≥2 symptomatic hemorrhages with a BSCM presenting to the pial surface (3). It is challenging because of the critical anatomy and potential risks of neurological or vascular injury. The advantages and risks of each surgical approach should be weighed carefully and chosen properly.

So far, with the advance of endoscopic techniques, eight cases of endoscopic transnasal approach to cavernous malformations have been reported in literatures. Here we present a case of midline ventral pontine CM that was resected through an endoscopic endonasal approach, which provides a straightforward trajectory and minimal brainstem stretch.

## Case History

### History and Examination

**Figure 1:**
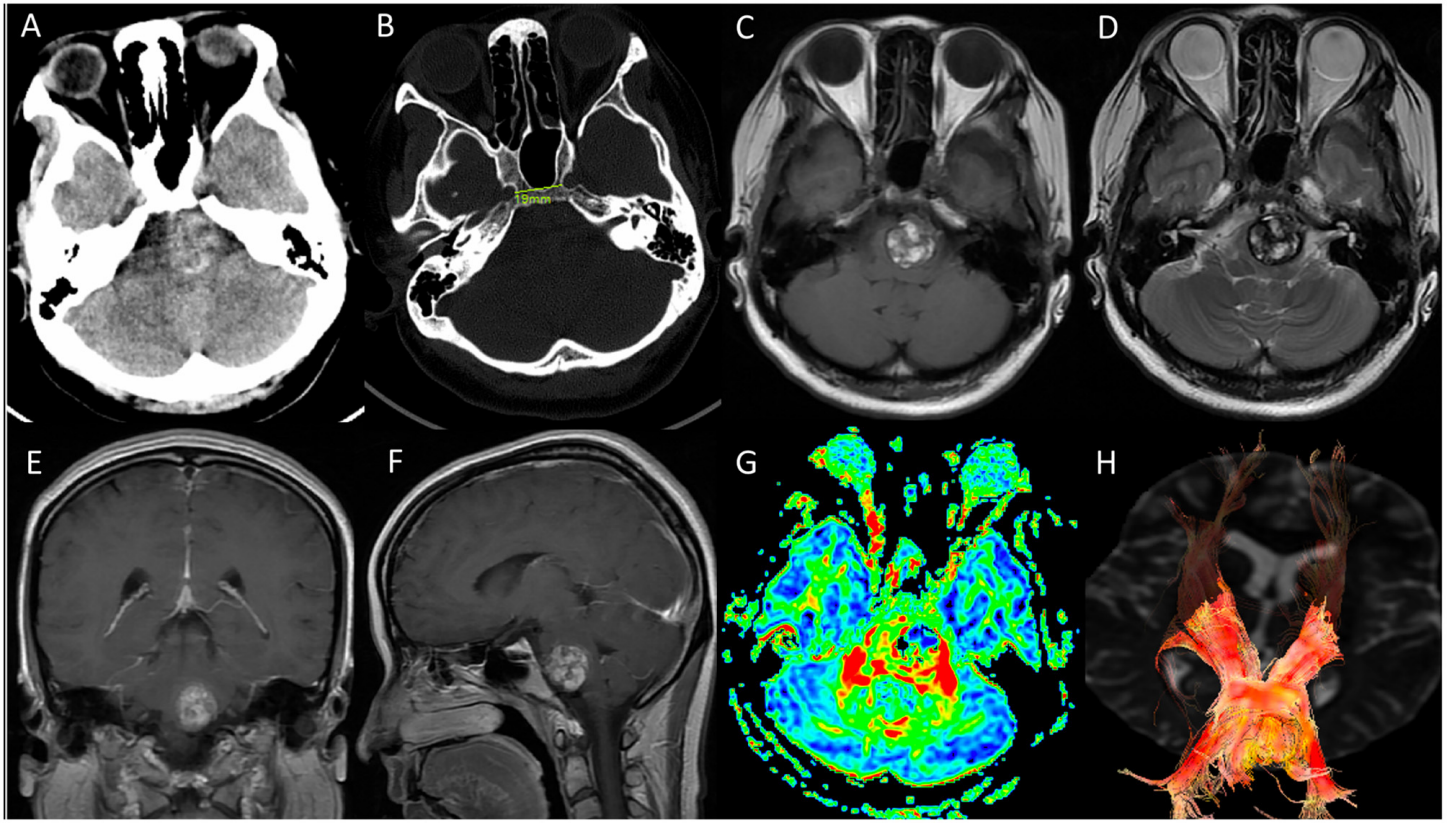
Preoperative imaging. **(A,B)** CT scan revealed a hemorrhagic occupying lesion in the left pons with acute blood products and indicated a distance of 19 mm between para clival carotid in bone window. The development and the degree of pneumatization of sphenoid sinus was suitable for endoscopic endonasal surgery; **(C–F)** Axial T1-weighted, T2-weighted, coronal and sagittal T1 postcontrast magnetic resonance image demonstrated a 2.3*2 cm ventral pontine CM with hemosiderin surrounded, and no significant enhancement of the CM was observed; **(G,H)** diffusion tensor imaging (DTI) showed the corticospinal tract was displaced laterally by the CM.

A 28-year-old woman was transferred to our neurosurgery department with a 1-month history of progressive right-sided hemiparesis, diplopia and hemiparethesia. No dysarthria, dysdipsia or facial numbness was presented. On neurologic examination, she was found to develop gradual right-sided hemiparesis with diminished strength of the right upper extremity (3/5) and right lower extremity (4/5). The lateral movement of her left eye was absent. A computed tomographic scan was obtained and was interpreted as demonstrating midbrain hemorrhage. Subsequent magnetic resonance image revealed a ventral pontine lesion eccentric to the left measuring 2.3^*^2 cm with lateral displacement of the corticospinal tracts (Figure 1). Given the patient's presentation and findings on imaging, pontine cavernous malformation was suspected. After an initial discussion of the potential risks of surgical resection vs. conservative management, she chose to undergo surgical resection. The CT scan demonstrated a distance of 19 mm between paraclival carotid and the development of sphenoid sinus which was suitable for endoscopic endonasal surgery (Figure 1).

### Operation

In this case, we elected to use an endoscopic endonasal transclival approach utilizing both nostrils and a 0° endoscope to resect the cavernous malformation (see Video 1). The patient was placed supine with her head fixed in a three-point Mayfield head holder with 10° of flexion and turned 15° toward the surgeon's side after general anesthesia, with her CT and MRI image registered to stereotactic guidance system to achieve the most accurate navigation in the high-risk area of the lesion in the brainstem. Intraoperative neurophysiologic monitoring of somatosensory evoked potentials and auditory evoked potentials were also included in our surgical setting. Lidocaine with 1:10,00,00 epinephrine was injected into the nasal mucosa and turbinates for hemostasis.

First, the middle turbinates were displaced laterally, and a vascularized nasoseptal flap was dissected and stored in the right choana inferiorly for subsequent closure. After both sphenoid ostia were opened, the anterior face of the sphenoid sinus was removed with a high-speed drill and the sphenoid mucosa was resected completely. Neuronavigation was periodically used to verify the trajectory to the lesion and the sellar floor and clivus are shown here just prior to drilling down the clivus to the level of the dura. Bleeding from the basilar sinus was controlled with SURGIFLO and an Aquamantys bipolar tip. Anatomic landmarks were used to identify the bilateral carotid arteries, the sellar floor, and the pharyngeal tubercles at the superior and inferior limitations of the bone resection, respectively.

After opening the dura and arachnoid, we identified the region where the cavernous malformation came closest to the surface of brainstem, which we used as our entry point. We used a microdissector, curettes, and biopsy forceps to free the cavernous malformation from the brainstem and remove it completely in piecemeal fashion. Care was taken to preserve the gliotic, hemosiderin-stained margin throughout the dissection. We then achieved hemostasis and inspected the cavity to ensure gross total resection prior to proceeding with closure. During the tumor removal, care was taken to identify and preserve the anterior inferior cerebellar artery, vertebral artery and the abducens nerve as illustrated here.

**Figure 2:**
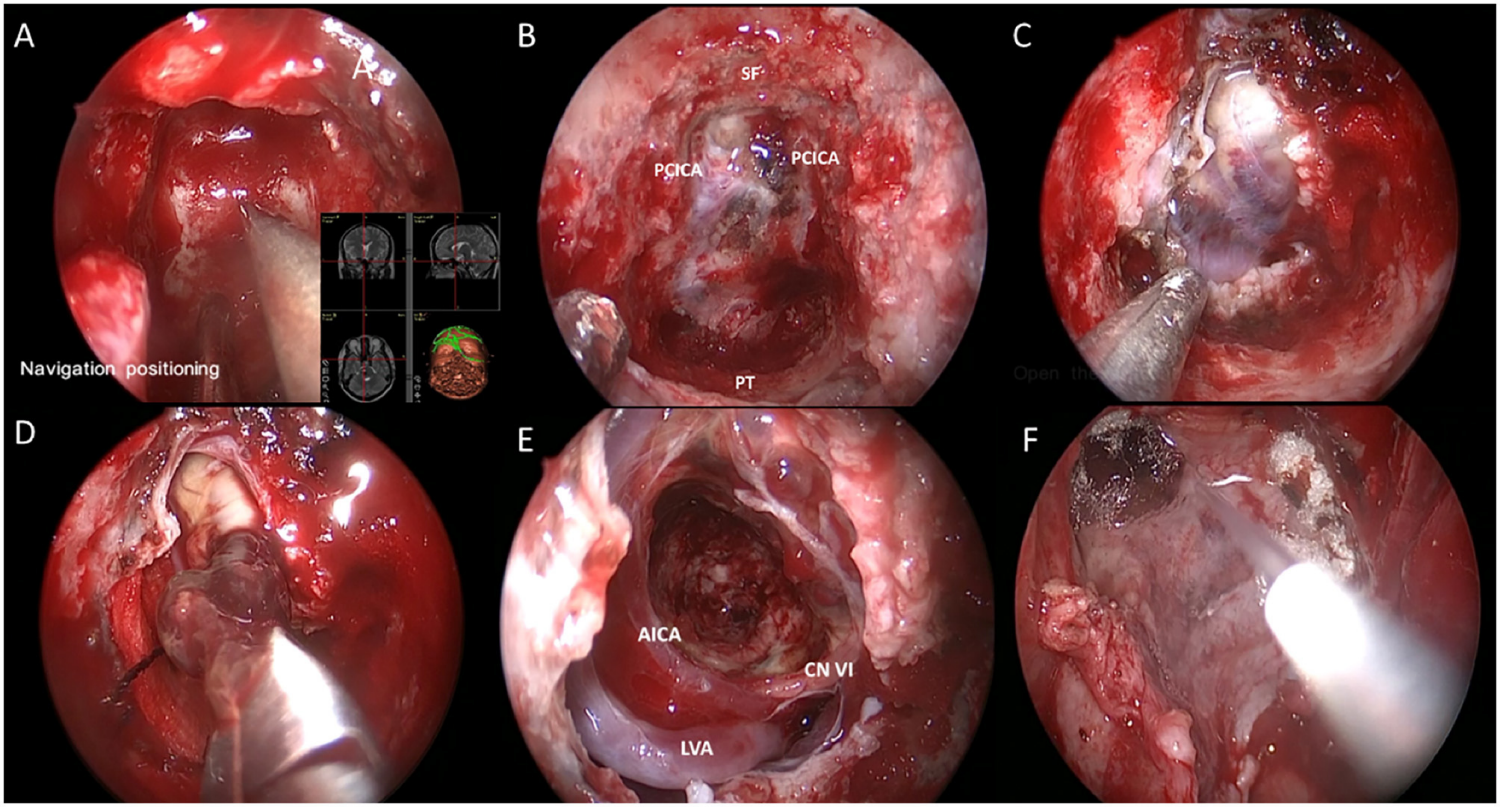
Endoscopic endonasal view of the intraoperative phases. **(A)** Intraoperative navigation locating surgical boundary; **(B)** Fully exposure of clivus. SF, PT and PCICA was illustrated; **(C)** Exposure of tumor after opening dura; **(D)** Dissection and removal of the cavernous malformation; **(E)** Intraoperative view through a 0° endoscope in the cavity; **(F)** Multi-layer reconstruction of skull base. Labeled structures: AICA, anterior inferior cerebellar artery; LVA, left vertebral artery; CN VI, cranial nerve VI; SF, sellar floor; PCICA, paraclival internal carotid artery; PT, pharyngeal tubercle.

To repair the skull base, we utilized a multi-layer reconstruction technique. First, artificial dura was inlayed to repair the durotomy defect. Next, autologous fat and fascia lata were harvested from the left anterolateral thigh and placed over the dural patch. We then reflected the nasoseptal flap over the repair construct and coated it with fibrin glue (Figures 2A–F). Finally, a Foley catheter microballoon was inserted and inflated to secure the nasoseptal flap and lumbar drainage was applied immediately after the operation.

### Postoperative Course

**Figure 3:**
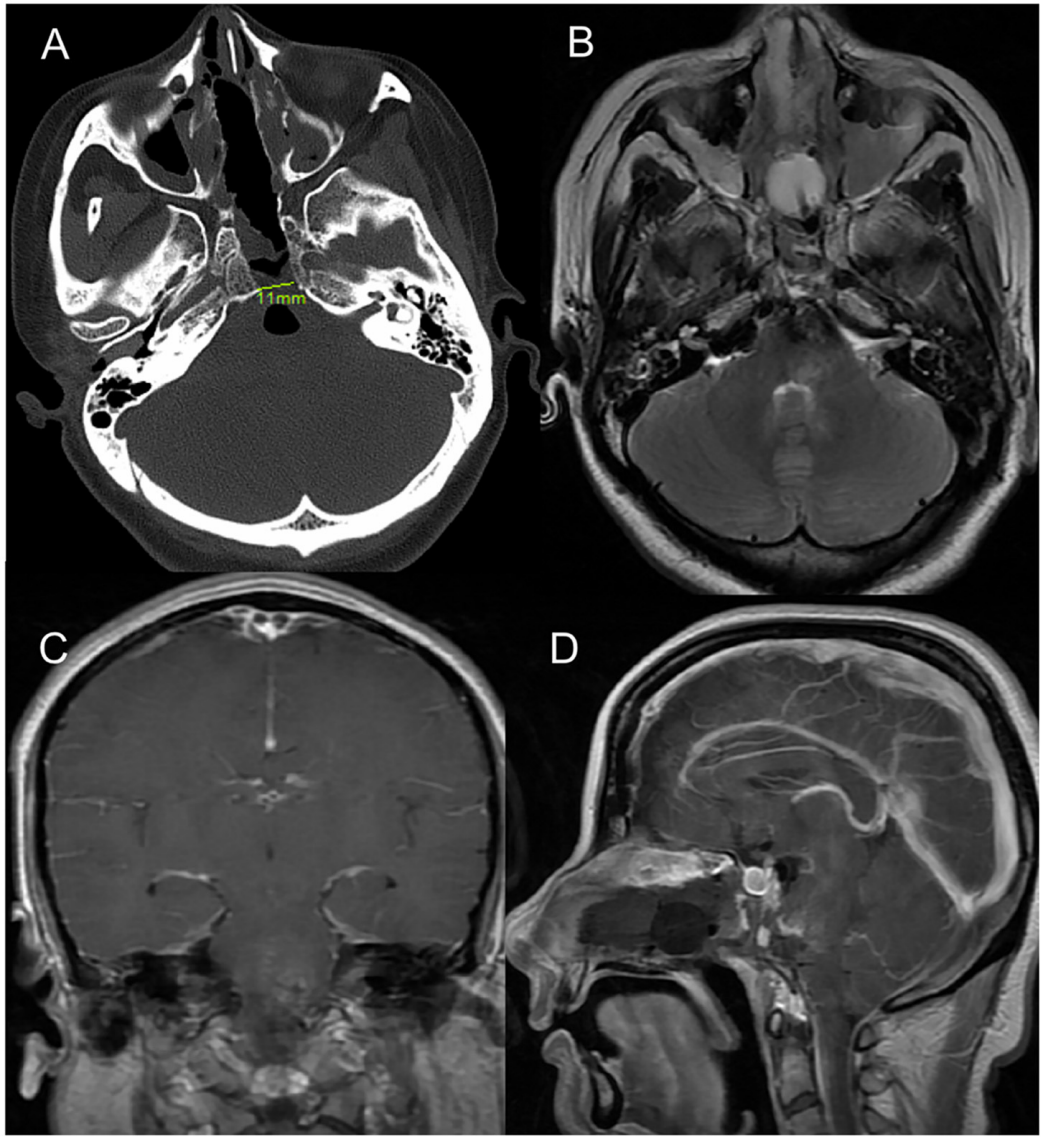
Postoperative imaging. **(A)** Post-operative axial CT image (bone window) indicating the drilled clival bone with a very limited opening of only 11 mm. **(B–D)** Axial T2-weighted, coronal and sagittal T1 postcontrast magnetic resonance image demonstrated no residual CM in ventral pons.

The post-operative MRI 4 days after surgery confirmed gross total resection of the cavernous malformation (Figure 3), and we did not observe any CSF leakage or worsening of the patient's presenting symptoms during the immediate recovery period. The Foley balloon was removed from the nose 5 days after surgery. And the drainage was removed on day 7. She was discharged 2 weeks after surgery. And the strength (4/5) of the patient's right extremities had improved by 1 month after surgery.

## Discussion

The indications and timing of surgery for BSCM are controversial. Giuliano et al. (4) reported that for symptomatic patients, if the lesion is close to the pial surface of the brainstem, surgery is recommended after first bleeding. Li et al. (5) recommended that when BSCMs grow to the pial surface of the brainstem, or acute hemorrhage leads to progressive neurological dysfunction, or lesions are larger than 2 cm and have a significant mass effect, surgery should be considered. For the timing of surgery, Giuliano et al. (4) preferred to perform surgery for BSCMs 2–3 weeks after the bleeding. Such a delay will allow the hematoma to be partially liquefied and separated from the surrounding brainstem tissue, thereby buffering and reducing surgical injury. But it should not take more than 1 month to perform the surgery, since at that time the organization and contraction of the hematoma can cause an adhesion of the lesion and the surrounding parenchyma, which increasing the risk of mechanical injury during operation.

The most ideal surgical approach to treat brainstem lesions should minimize the stretch of the brain and expose the lesion as much as possible for the treatment of cavernous hemangioma to reduce the postoperative complications. Several approaches can be used for the treatment of brainstem cavernous malformations, including the cerebral peduncle approach, presigmoid approach, extradural and intradural petrosal approach, sigmoid sinus or extended retrosigmoid approach, combined retrosigmoid approach and subtemporal approach. All these approaches can provide a path to the anterior and lateral brainstem. However, for the ventral midline lesions, which is close to the pontine surface, none of these approaches is appropriate. The lateral or posterolateral approach, especially transpetrosal approach, requires extensive bone flap and stretch of cranial nerves and vessels. To make it worse, these approaches do not provide a direct manipulation angle for ventral pontine BSCMs. In addition, a large study reported that surgery via the lateral approach for BSCMs at pontomedullary junction would cause a stretch of the ventral longitudinal corticospinal tract, resulting in disability in 30% of patients (6).

Endoscopic endonasal approach has its own advantages over other approaches. The nasal cavity and sinus space provide a natural pathway for surgeons to reach the skull base directly, avoiding any incision of the scalp or maxillofacial shift and minimizing retraction of cranial nerves and blood vessels. The neurological anatomy will be preserved as much as possible. It is critical in the concept of modern minimally invasive surgery. In recent years, with the popularization of neuronavigation and development of skull base reconstruction techniques, endoscopic endonasal approach has been applied to more skull base lesions than ever.

Reisch et al. (7) reported two cases of microscopic transoral transclival approach to ventral brainstem cavernous malformations for the first time in 2001. With the improvement of endoscopic techniques and instruments, more skull base lesions can be removed via endoscopic endonasal approach, including lesions in the ventral aspect of the brain stem, which can be exposed and resected via transclival approach. Kimball et al. (8) reported the first endonasal transclival approach to ventral pontine cavernous malformation in 2012. And He Shi-ming et al. (9) applied the endoscopic endonasal transclival approach for the resection of a hemorrhagic, symptomatic CM in ventral mesencephalon in 2016. So far, nine cases of endoscopic transnasal approach to cavernous malformations have been reported in literatures (Table 1) (8–16).

**Table 1:** Comparison of reported cases of endoscopic endonasal transclival resection of BSCMs.

**Case**	**Authors and Year**	**Age**	**Location**	**Dimensions(mm)**	**Presenting symptoms**	**Extent of Resection**	**Nasalseptal flap**	**Precautionary lumbar drainage**	**Complications**	**Postoperative deficts**
1	Kimball et al. (8)	59	Ventral midpons	20 × 20 ×20	bilateral facial numbness, diplopia, left hemiplegia, right CN VI and VII palsy	Gross total	Yes	No	CSF leak	Facial weakness, CN VI palsy and muscle strength in left extremities improved
2	Sanborn et al. (10); Nayak et al. (11)	17	Ventralmedial pons	17 × 12	Headache, facial numbness, tingling, left-sided hemiparesis, right sixth nerve palsy and dysphagia	Total	Yes	Yes, for 48 h	CSF leak	Left-sided hemiparesis, facial nerve weakness, bilateral restricted horizontal gaze
3	Nayak et al. (11)	60	Ventromedial cervicomedull ary junction	8 × 9 ×10	Right-sided hemiparesis, CN intact	Total	Yes	/	/	Symptoms remained unchanged in the immediate postoperative period and at 3-month follow up.
4	Linster and Oertel (12)	29	Ventromedial brainstem	20 × 18 ×22	numbness and tingling of the right extremities, loss of fine motor control of the right hand, transient diplopia and headache	Total	No	Yes, for 5 days	None	None
5	Dallan et al. (13)	15	Ventralmedial pons	10 × 10	Acute onset of severe cephalalgia, right cranial nerve VI, VII, and VIII palsies	Subtotal	Yes	No	None	None
6	He et al. (9)	20	Ventromedial mesencephalon	12 × 17	Headache, nausea, and vomiting, left-sided hemiparesis, absent lateral and medial left eye movements and pupillary light reflex	Total	Yes	Yes, for 6 days	None	None
7	Gómez-Amador et al. (14)	29	ventral pontine	18 × 26 ×29	Headache, nausea, diplopia, somnolence, facial palsy, dysarthria, dysphonia, dysphagia, and left hemiparesis	Total	Yes	No	None	Improved left leg strength and CN VI and VII function
8	Alikhani et al. (15)	26	Left medulla oblongata	15 in greatest diameter	Imbalance, swallowing difficulty, right hemibody weakness	Total	Yes	No	None	Improved right hemibody weakness
9	London et al. (16)	51	Left pontine	/	Diplopia, dysphagia, and ataxia	Total	Yes	Yes, for 5 days	None	Aseptic meningitis for 2 weeks
10	Present case	28	Ventral pontine	23 × 20	Right-sided hemiparesis, diplopia and hemiparethesia	Total	Yes	Yes, for 7 days	None	Improved right extremities strength

The result of these 10 cases (nine previously reported and one present) are promising. The total resection rate was 90.0% (9/10), which was close to the total resection rate of BSCM obtained by meta-analysis (91%) (17). Of these cases, three patients had no clinical symptoms or neurological deficits, and the clinical symptoms of seven patients had significantly improved. The total operative mortality was 0%, compared with the 1.5% in meta-analysis (17). The incidence of CSF leak was 22.2% (2/9), while one of which applied lumbar drainage and the other did not. In comparison, no CSF leak was observed in the rest seven cases, while four case applied lumbar drainage, and three cases did not. This may imply that the incidence of CSF leak is little related to the application of lumbar drainage.

The biggest concern with endoscopic endonasal approach to intradural lesions is cerebrospinal fluid leakage. Despite the evolution of endoscopic techniques and equipments, CSF leakage remains as the most common postoperative complication of endoscopic surgery for skull base lesions. The incidence of CSF leakage in the published literatures is lower than that in clinic. To prevent the occurrence of CSF leakage, different concepts and methods are recommended by different neurosurgeons. Linsler and Oertel (12) used autologous umbilical fat grafts to repair dura mater and all patients underwent continuous lumbar cistern drainage for at least 5 days after endoscopic endonasal surgery. In addition, they merged CT/MRI image for navigation to minimize the size of the bone flap and opening dura. In their particular case of endoscopic transclival approach to brainstem CM, the diameter of the bone flap was only as small as 7 mm, and nasal septal mucosal flaps are not needed. However, Gomez-Amador et al. (14) recommended that the application of intralayer artificial gasket suture and vascular pedicle flap of nasal septum for multilayer reconstruction can significantly reduce the incidence of postoperative cerebrospinal fluid leakage. And continuous lumbar cistern drainage was not necessary.

### Experiences From This Particular Case

1. Careful preoperative evaluation: Several sequence of MRI have been conducted before the surgery for a comprehensive evaluation. The distribution of brainstem corticospinal tract can be demonstrated in diffusion tensor imaging (DTI), which will be useful for choosing an appropriate surgical approach.
2. Using multi-modal technology, including intraoperative Doppler ultrasonography, magnetic resonance navigation and electrophysiological monitoring, to achieve a precise positioning of the lesion and demonstrate the anatomy of tumor and its surrounding structure, which makes the operation much safer.
3. Surgeons must be familiar with the anatomic structure of skull base with experienced surgical skills and adequate endoscopic instruments. That is what it takes to perform a successful endoscopic endonasal surgery for brainstem lesions.
4. Considering the complexity of abundant vascular nerve nucleis in ventral brainstem at the level of clivus, bony structure and dura should be exposed adequately under the condition of safety. Then the tumor can be totally removed with the anatomy and function of the normal brainstem preserved.
5. Effective reconstruction of the skull base: We use multilayer skull base reconstruction technique (fat for the cavity, fascia and pedicled vascular nasal septum flap covering the defect as the “sandwich”) and lumbar cistern drainage to achieve a satisfactory outcome. For this particular patient, no postoperative CSF leakage was obtained.

## Conclusions

Here we present a successful endoscopic endonasal transclival resection of pontine CMs. Compared with conventional craniotomy, this approach have its own advantages, such as minimal brainstem transection, negligible cranial nerve manipulation, and a direct trajectory to the ventral brainstem. However, it requires delicate and experienced endoscopic surgical skills and abundant instruments to use this approach. And the risk of CSF leakage is still the major concern of neurosurgeons, which should be prevented with proper skull base reconstruction techniques.

## Data Availability Statement

The original contributions presented in the study are included in the article/Supplementary Material, further inquiries can be directed to the corresponding author/s.

## Ethics Statement

The studies involving human participants were reviewed and approved by the Ethics Committee of the Second Affiliated Hospital of Zhejiang University Medical College. The patients/participants provided their written informed consent to participate in this study.

## Author Contributions

JZ and YH contributed conception and design of the study. AS organized the figures. XD wrote the first draft of the manuscript and critically revised the final manuscript draft. XW wrote sections of the manuscript. All authors contributed to manuscript revision, read, and approved the submitted version.

## Conflict of Interest

The authors declare that the research was conducted in the absence of any commercial or financial relationships that could be construed as a potential conflict of interest.
